# Neural correspondence to spectrum of environmental uncertainty in multiple-cue probability judgment system with time delay

**DOI:** 10.3389/fncom.2025.1595278

**Published:** 2025-07-17

**Authors:** Yoo-Sang Chang, Younho Seong, Sun Yi

**Affiliations:** ^1^Department of Industrial and Systems Engineering, North Carolina A&T State University, Greensboro, NC, United States; ^2^Department of Mechanical Engineering, North Carolina A&T State University, Greensboro, NC, United States

**Keywords:** decision-making, electroencephalogram, expected uncertainty, unexpected uncertainty, cognitive process, multi-agent system, time delay

## Abstract

Despite state-of-the-art technologies like artificial intelligence, human judgment is critically essential in cooperative systems, such as the multi-agent system (MAS), which collect information among agents based on multiple-cue judgment. Human agents can prevent impaired situational awareness of automated agents by confirming situations under environmental uncertainty. System error caused by uncertainty can result in an unreliable system environment, and this environment affects the human agent, resulting in non-optimal decision-making in MAS. Thus, it is necessary to know how human behavior is changed to capture system reliability under uncertainty. Another issue affecting MAS is time delay, which can delay agent information transfer, resulting in low performance and instability. However, it is difficult to find studies on the influence of time delay on human agents. This study is about understanding the human decision-making process under a specific system reliability environment by uncertainty with time delay. We used concepts of expected and unexpected uncertainty to implement reliability of the system usage environment with three types of time delay conditions: no time delay, regular time delay, and irregular time delay conditions. We used electroencephalogram (EEG) for human cognitive neural mechanisms in multiple-cue judgment systems to understand human decision-making. In the reliability of system usage environment, the unreliable system environment significantly creates less memory load by less utilization of system rules for decision-making. In terms of time delay, delayed information delivery does not significantly affect memory load for decision-making.

## 1 Introduction

Humans play an essential role in multi-agent systems (MASs), even though artificial intelligence technologies are being developed. MAS is a cooperative system based on the interaction among agents to solve problems (Balaji and Srinivasan, 2010). A representative example of MAS is the defense system with autonomous agents, such as unmanned ground vehicles (UGVs) or unmanned aerial vehicles (UAVs). The MAS-based defense system is operated by a multiple-cue judgment system by human agents based on obtained information (multiple cues) for appropriate decisions in a dynamic environment (Sokolowski, 2003).

The human agent is essential to MAS because it makes decisions based on evaluating the current system’s performance. The human agent inspects the system performance by comparing information from autonomous agents and real-world results to check whether the system shows valid performance for the goal. The system performance can fluctuate due to system errors such as communication network or sensor errors in a dynamic environment. The fluctuating system performance can cause invalid system performance by receiving and transmitting invalid information under uncertain environments. The human agent makes decisions after examination of system performance, whether it can make valid or invalid system performance by checking to make decisions for aimed output. Based on these experiences of system usage, the human agent tries to avoid system failure due to impaired situational awareness caused by system error of autonomous agents in MAS (Tweedale et al., 2007).

### 1.1 Human decision-making under environmental uncertainty

Despite inappropriate decision-making avoidance by the human agent, environmental uncertainty affects the MAS performance because of an unreliable system usage environment. MAS operates in dynamic environments with unexpected situations. The unstable environments cause system errors, such as communication network errors among agents or physical sensor errors due to geographic or weather conditions (Yiu et al., 2022). The incorrect information due to the unstable environments causes unreliable system usage contexts, resulting in loss by experiences of invalid system performance to human agents.

There are two reasons for the non-optimal choice of uncertainty from the neurological perspective. The first reason is the neural state changes for every decision-making trial, such as the atmosphere under a specific environment. The second reason is the noise of input observation, such as incorrect information reception (Geng et al., 2020). For the characteristics of sensitive neural states of humans, the non-optimal decision-making can be worse by the negative effect of unreliable system usage experiences. Thus, studies of the human cognitive process are needed to deal with the impact of environmental uncertainty in MAS with human agents’ decision-making characteristics for high-level MAS.

### 1.2 Decision-making under time delay of information delivery

**Figure 1:**
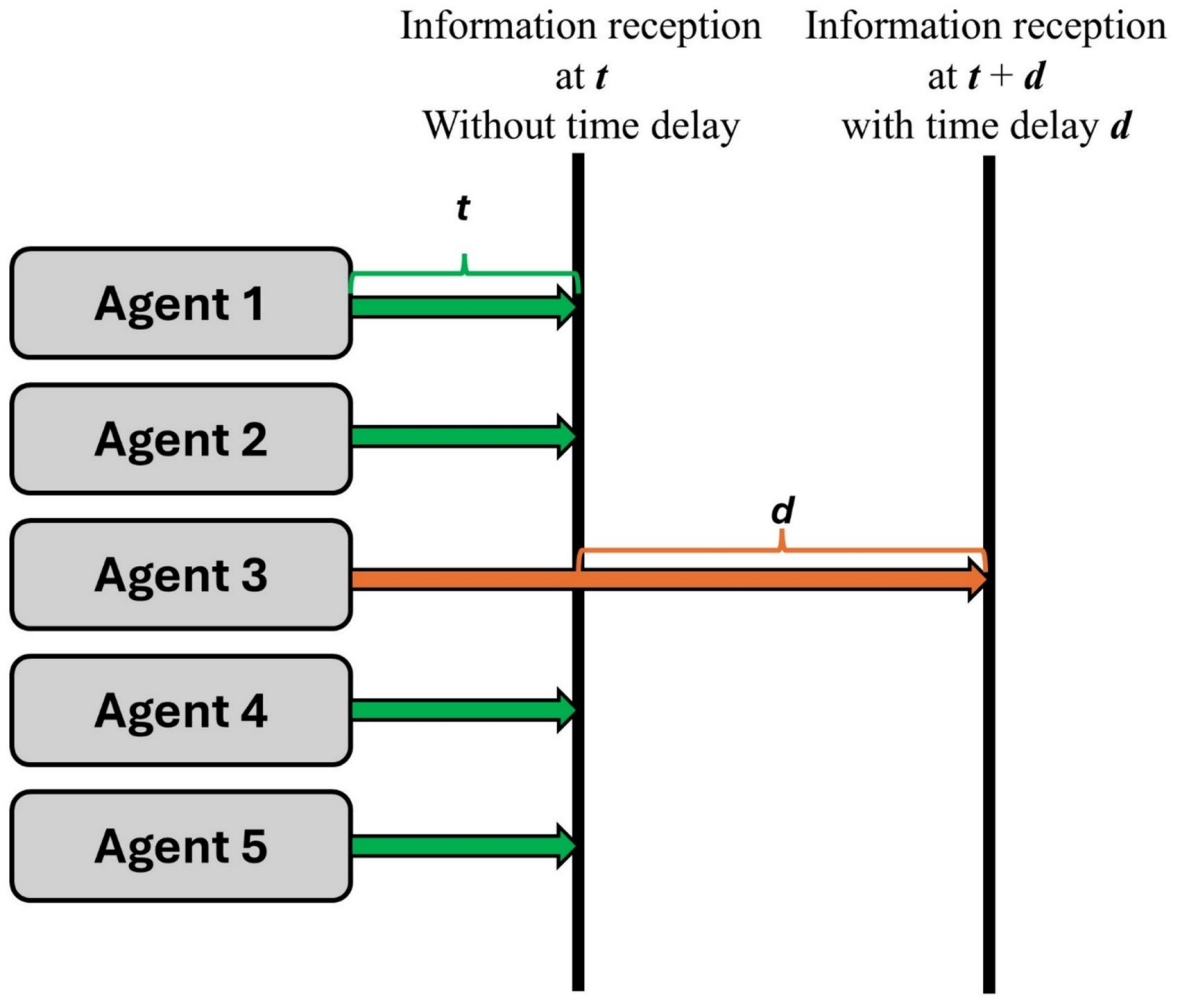
Time delay concept in information delivery.

Time delays can influence the performance of MAS. The communication network in MAS is operated based on a distributed network system to control cooperative autonomous agents with information sharing. During information sharing, a time delay occurs when the communication network system has issues, such as loss of packet, uncertain system mode, or unexpected interference among agents in MAS (Zhang et al., 2018). The time delay by communication system errors can cause low system quality, resulting in performance instability due to system degradation (Sumpter et al., 2019) and serious problems, such as system breakdown (Zhang et al., 2018). There are some studies on improving communication network performances of MAS performances considering time delay in terms of mathematical models among autonomous agents (Li et al., 2012; Li et al., 2018; Trentelman et al., 2013). Sumpter et al. (2019) considered trust aspects based on topology and time delay for agent communication to build a mathematical MAS model. In contrast, it is difficult to find studies about human cognitive characteristics under time delay in MAS. This study focuses on time delay regarding information reception by the human agent. Figure 1 shows the concept of time delay in information delivery.

In this study, the time delay is assumed to mean that human agents can receive at the time “t” if there is no delayed information delivery from each agent. In contrast, they will receive information at time “t + d” if they receive one information with “d” time delay after receiving other information at the time “t.” Based on the experience of time delay, we expect an insight into how the human cognitive process is performed to deal with the delay in information delivery in MAS.

### 1.3 Expected and unexpected uncertainties

**Figure 2:**
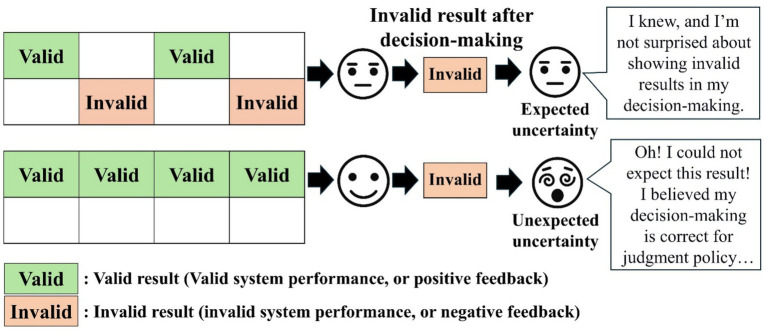
The explanation of expected and unexpected uncertainty concepts.

We used concepts of expected and unexpected uncertainties to implement unreliable system usage environments by uncertainty, as suggested by Yu and Dayan (2005). The expected and unexpected uncertainty is made using stimulus–response–outcome (S-R-O) rules. The agent can get benefits or losses (outcome) through learning about the relationship between stimulus and response. For example, they see different colors of balls, such as green and blue. These balls are stimuli (S) information before decision-making. This decision-making, choosing one color ball, is response (R), which leads to outcomes (O). According to the response, the outcome (O) is getting (positive) or loss (negative) of one dollar. For example, if they choose green ball and the outcome is getting one dollar, it gives positive feedback. Thus, they can learn that green balls are associated with benefits. In contrast, if they lose one dollar by choosing the blue ball, the decision policy of negative feedback is associated with the blue ball. The experience ratio of positive or negative outcomes determines the expected and unexpected uncertainty. The expected uncertainty can occur when they observe a lot of negative feedback, such as invalid outcomes. In contrast, unexpected uncertainty can occur through many observations of positive feedback, such as valid outcomes. So, the expected uncertainty is knowing that the specific judgment system or policy is unstable and unreliable in getting valid results by observing many invalid results (e.g., 50% invalid system performance observations). If the decision-maker is under the expected uncertainty, they will not use the current decision policy to explore another policy for their benefit. In contrast, unexpected uncertainty gives intense violated experiences by less invalid result observation (e.g., 10% invalid system performance observations). Unexpected uncertainty occurs in a reliable decision-making environment, so they will exploit current decision-policy rather than decision-policy under expected uncertainty (Bland and Schaefer, 2012). Based on the concept of expected and unexpected uncertainty, we set up the experience of environmental uncertainty by showing intended invalid system performances to build reliable or unreliable system usage performances based on expected and unexpected uncertainty concepts, as shown in Figure 2.

### 1.4 Relationship between trust in system and expected and unexpected uncertainties

The expected and unexpected uncertainties are related to trustworthiness in automated systems with humans. In automation, trust refers to the attitude of an agent to use or not use an automated system (Lee and See, 2004). Trust in automation is a critical issue. For example, suppose human agents would not use information from an automated system, such as changing the railroad to avoid a collision with a forward train despite correct information because of distrust of the system. In that case, they will not change the railroad. Then, collisions happen, resulting in system breakdown and loss of lives (Dzindolet et al., 2003). Thus, to prevent the disuse of the system in appropriate situations, trustworthiness should be measured by human agents.

There are studies that seek to understand human agents’ cognitive states regarding the experience of trust or distrust by adjusting invalid results. Oh et al. (2020) implemented invalid result stimulus by incorrect action, as invalid system performances. For example, the participants decided to drive to the third lane because the vehicle existed in front of the first and second lanes. In this case, the invalid result is that the vehicle was moved to the first lane, resulting in a collision by invalid system action even though they controlled vehicles driving to the third lane. Choo and Nam (2022) suggested a classifier for trust and distrust situations by showing invalid results based on given information to solve the problem with decision-making policy.

In a study of expected and unexpected uncertainty, Kogler et al. (2017) experimented with understanding cognitive states in expected or unexpected uncertainty using invalid results. They control the ratio of invalid results to make expected and unexpected uncertainty situations. For example, a cue, such as a star, circle, or triangle, is given for decision-making. The valid result is shown with rewards if they press the buttons related to each shape. For example, the reward (money) is increased by pressing the star shape button when they see the star shape. The invalid result is a loss of points by pressing the star shape when the cue is also a star shape. The expected uncertainty comprised 50% of invalid results, and the unexpected uncertainty situation comprised 20% of the invalid results after decision-making. Like the studies of trust in automation and expected and unexpected uncertainty, trustworthiness or expected and unexpected uncertainty depends on the ratio of invalid results. Therefore, this study focuses on understanding human cognitive states under different reliability levels of system usage environment (decision-making environment) under expected and unexpected uncertainty situations in multiple-cue judgment systems with a time delay of information delivery.

### 1.5 Electroencephalogram

Electroencephalogram is a measure of minute brain signals from the scalp. There are two types of EEG devices. First is invasive EEG, which measures the signal through implanted devices with brain surgery. The second is a non-invasive EEG device that wears a cap with electrodes to record EEG data. Non-invasive EEG devices show lower EEG data quality than invasive EEG devices, but safety is much better than invasive EEG devices because there is no surgery to record the signal (Ahmadian et al., 2013). Non-invasive EEG data records brain signals through electrodes. These electrodes are mainly located on five different lobes, such as frontal (F), central lobe (C), parietal lobe (P), temporal lobe (T), and occipital lobe (O). The electrode layout is up to the study goal, where they want to know how the neural activity operates (Kumar and Bhuvaneswari, 2012; Lim et al., 2018).

The measured signals are used to understand neural activity by analyzing the amplitude change in the signal. EEG data is interpreted using different analysis domains. The first domain is the time domain based on the amplitude of potential value in time windows. The representative time domain analysis is the event-related potential (ERP), which is an analysis of amplitude peaking when a human experiences an event (stimulus) (Du et al., 2013). The second is frequency domain analysis through a transformation from a time window to a frequency window. The frequency domain analysis is used to know brain states, such as sleep, relaxation, or anxiety according to the different frequency bands (Firoz et al., 2022). The last analysis domain is the time–frequency domain, which is used to understand brain activity through frequency change for each time window (Al-Fahoum and Al-Fraihat, 2014). This study used a non-invasive EEG device with ERP analysis as time domain analysis.

### 1.6 Event-related potential studies in human decision-making

For analysis of neural states under uncertainty in a multiple-cue judgment system, we used the ERP. ERP is a temporal analysis that measures amplitude (potential value) in a given the time window. Generally, neural states are interpreted by positive or negative peaking of amplitude from the onset, which is getting feedback timing from the event. The representative ERP pattern is P300, which shows positive peaking after 300 ms from the onset. The P300 patterns have been interpreted in cognitive states. For example, a higher P300 amplitude means the mental state is superior. In contrast, lower P300 amplitude occurs when the cognitive state is relatively weak, such as alcohol, drug, or nicotine dependence (Sur and Sinha, 2009).

ERP analysis is used in trust in human decision-making. Long et al. (2012) analyzed ERP for trust choice. The ERP pattern was shown for “gain,” as a trustful choice, and “no gain,” as a distrustful choice. In this study, feedback-related negativity (FRN) and P300 patterns appeared. FRN is showing a negative peak amplitude from 200 to 300 ms after onset. P300 amplitude, as the P300 effect, is increased for trustful and distrustful choices. FRN effect is decreased in distrustful choice. de Visser et al. (2018) measured trust in different agent algorithms. The trust or distrust performances were significantly distinguished by observational error-related negativity (oERN), showing negative peaking after about 44 ms from the onset, and observational error positivity (oPe), with positive peaking after about 150 ms from the onset. The one is shown by detecting unconscious errors, and oPe is shown by recognizing errors. Trust states were measured when humans have conversations with a chatbot in e-commerce by showing P2 (P200), showing positive or negative peaking approximately 200 ms after onset, and late positive potential (LPP) showing positive peaking approximately 600 ms after onset. P2 is shown under low-level performance of the system, and LPP is shown under conscious control with sustained attention (Wang et al., 2023).

ERP studies have been conducted on expected and unexpected uncertainty. Kogler et al. (2017) performed an ERP analysis to determine how neural states change when humans are under expected and unexpected, uncertain situations by giving positive and negative feedback in gambling experiments. FRN, showing negative peaking approximately 200–300 ms after onset, was shown when they saw negative feedback to their decision-making under uncertainties. P300 pattern appeared in positive feedback under the expected and unexpected uncertainty. Boelaert (2022) performed ERP studies by decision-making based on multiple pattern cues under expected and unexpected uncertainties. P300 and N2pc, showing negative peaks approximately 180–300 ms after onset. N2pc is one of the N2 (N200) patterns, and it can appear when humans pay attention to distractors instead of targets.

ERP studies show neural states under trust and distrust, or expected and unexpected uncertainty. However, it is difficult to find ERP studies in the multiple-cue judgment system, such as MAS, with time delay issues for information reception. This study focused on how neural states operate under expected and unexpected uncertainty situations with a time delay in information delivery in the multiple-cue judgment system.

This study is about understanding the cognitive process of the human agent based on decision-making under uncertainty in the multiple-cue judgment system, such as MAS. Based on concepts of expected and unexpected uncertainties, the uncertainty is implemented by showing the frequency of invalid system performance after decision-making. Also, a time delay is added to investigate how this issue affects human decision-making by multiple cues (information) with time delay. An EEG measures neural correspondence after experiencing the multiple-cue judgment system. This study will give insight into how an unstable and unreliable decision-making environment under uncertainty affects the human agent’s decision-making process with time-delayed information transmission for developing high-level MAS.

## 2 Method

### 2.1 Experimental setup for unreliable system performance environment

**Figure 3:**
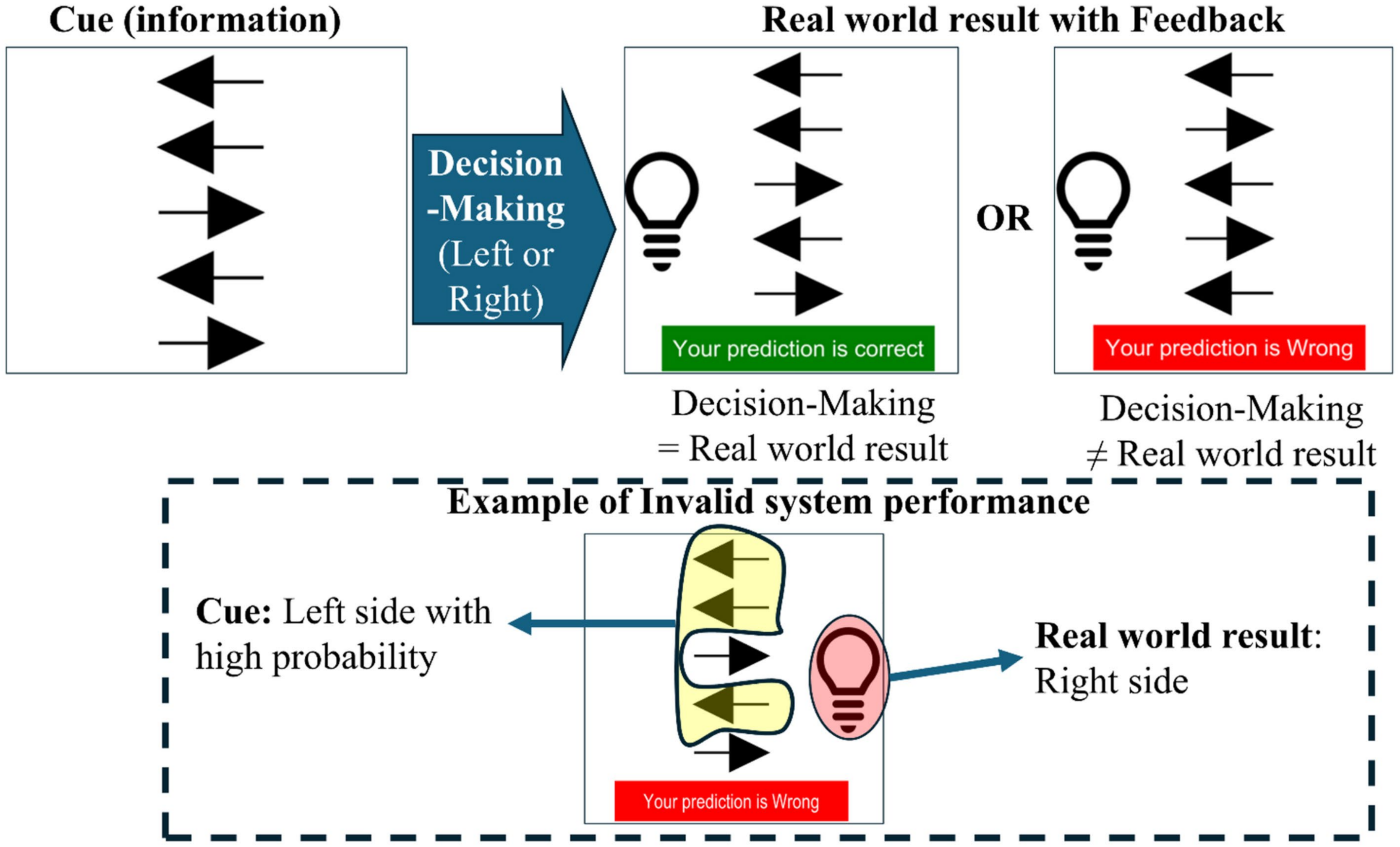
Experiment design for the valid and invalid results according to decision-making.

In this study, the unreliable system usage environment is comprised of a ratio of invalid system performances based on multiple cues and a real-world result in decision-making. The five agents’ cues have information on where the bulb will be located between left and right. The agent can detect the bulb’s location and transmit information to a human agent. The real-world result shows the bulb’s location with feedback, such as correct or wrong prediction based on decision-making with cues, as shown in Figure 3.

**Figure 4:**
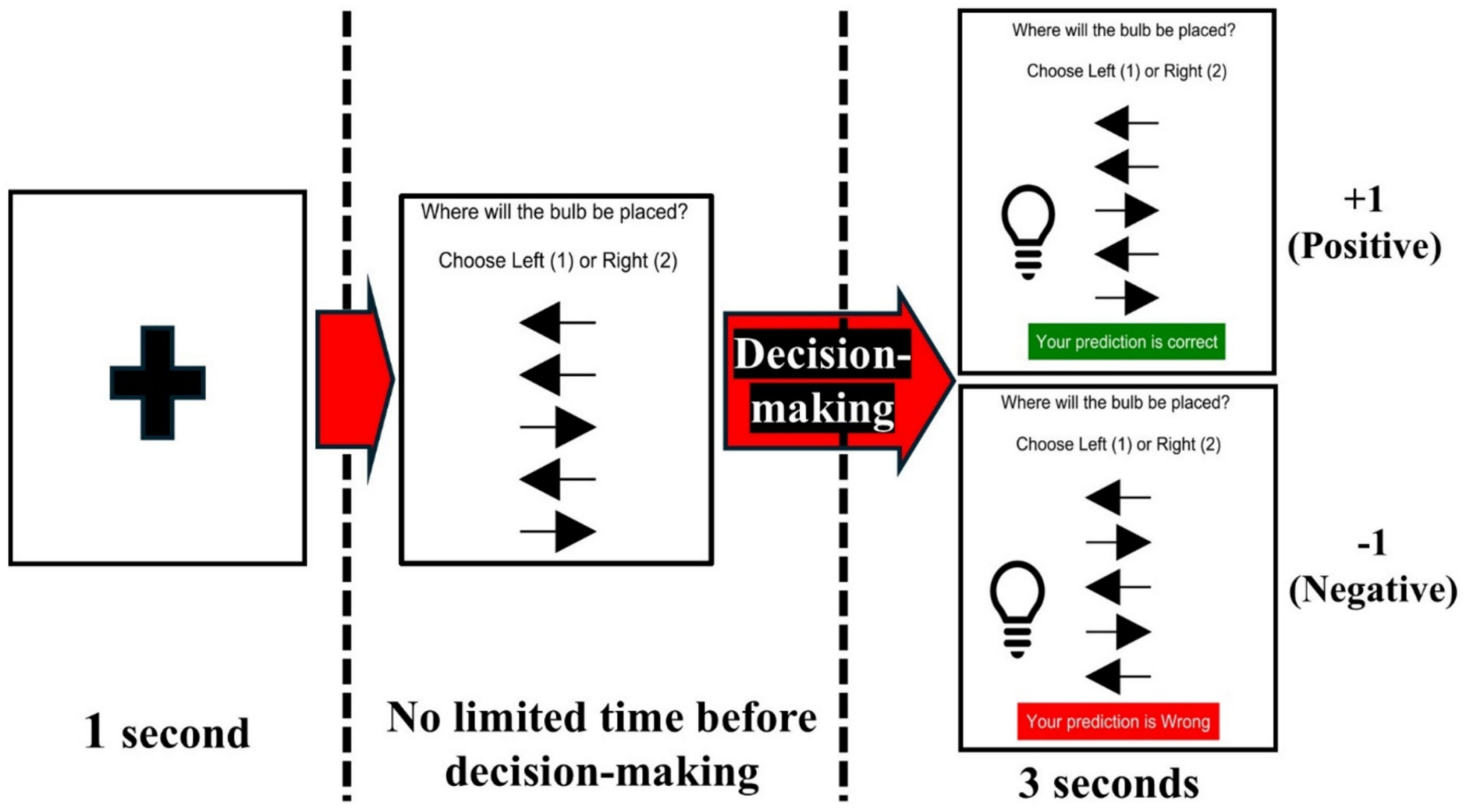
Experiment process in one decision-making trial.

Invalid system performance means that it is not the same between given multiple cues from each agent and real-world results after decision-making in this study. For example, suppose that the real-world result is that the bulb is located on the right side when three out of five arrows point out left in the cue section. In this case, an invalid system performance situation is shown, which can result in the wrong consequences for the bulb location in the real world for the system aim. In the experiment process for each trial in Figure 4, fixation is shown for 1 s to refresh. Then, multiple cues (arrows) are shown. For decision-making based on multiple cues, we let decision-makers know they should press keypad “1” if they think the bulb will appear on the left side and that they should press keypad “2” if they predict the bulb will be on the right side.

The expected and unexpected uncertainty situation consists of the different ratios of invalid system performances to build three different system usage environments. The invalid system performance is when multiple cues and real-world results are different. The first is the perfect system environment (PF) with no invalid system performances. Second is the expected uncertainty environment (EX) with a 50% ratio of invalid system performances. Finally, the unexpected uncertainty environment (UX) is a 10% ratio of invalid system performances. There are 60 decision-making trials for each environment. The invalid system performance is manipulated intently for reliable or unreliable system usage environments.

### 2.2 Experimental design for time delay

**Figure 5:**
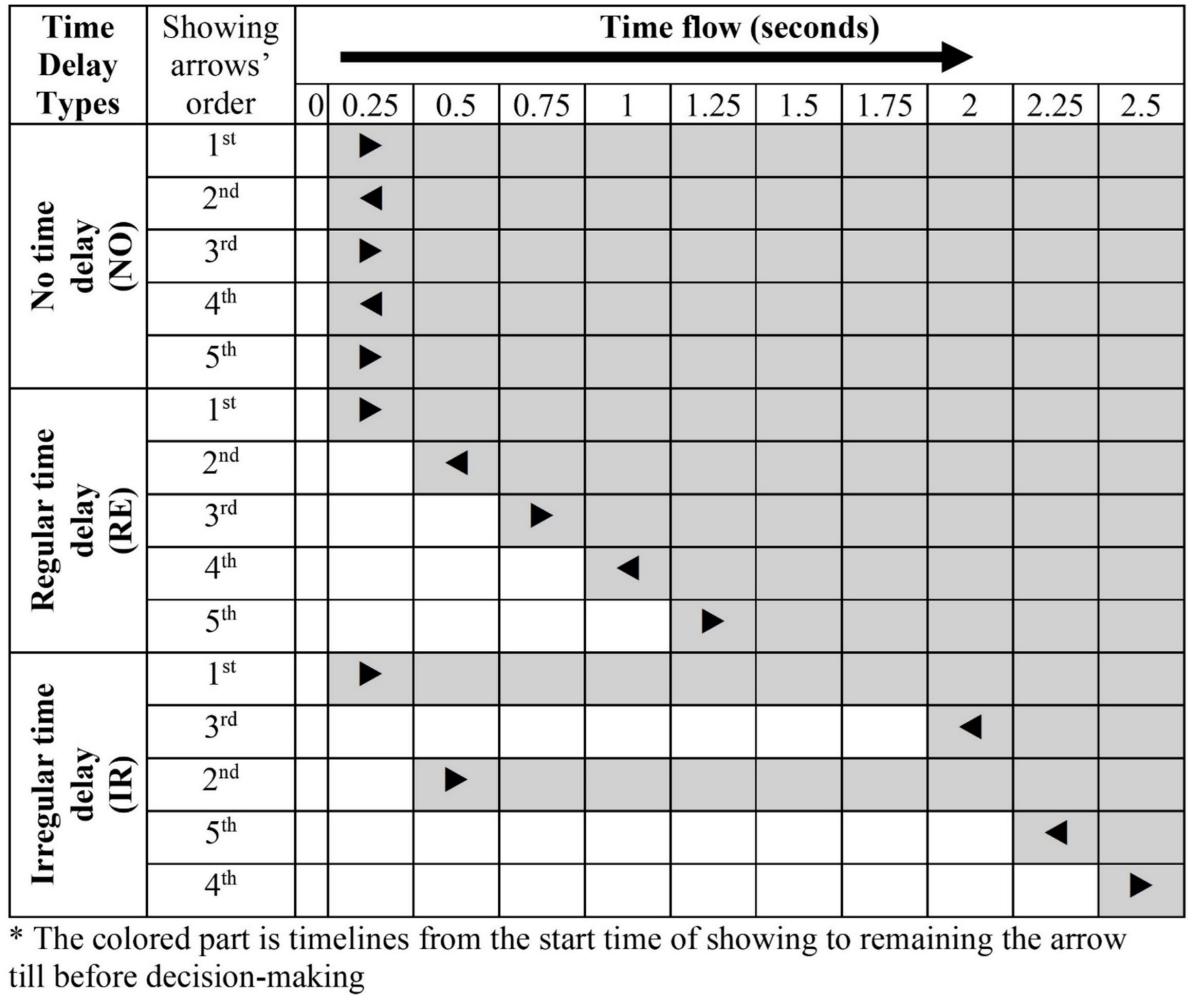
Experiment with the design of time delay conditions by time flow.

The time delay is implemented by showing each arrow with specific interval times with three types of conditions. The first is no time delay (NO), showing all five arrows simultaneously. The second is regular time delay (RE), showing the four arrows with 0.25-s intervals after showing the first arrow. The last is the irregular time delay (IR), showing one arrow after 1.5 s, while the other four arrows are shown with a 0.25-s delay. The first arrow is shown after 0.25 s from the empty display for all conditions, and the arrows are not shown simultaneously in RE and IR conditions. The order of the arrows shown is random, not shown from top to bottom, as shown in Figure 5.

The experiment is conducted in nine conditions combined with three types of system usage environments and three types of time delay situations, as shown in Table 1. We first experimented, showing NOPF. After the NOPF condition, the other eight conditions were randomly presented to participants. This study used PsychoPy (2023.2.3) as an experiment tool based on Python code.

**Table 1:** The number and ratio of invalid system performance to build three different system usage environments (uncertainty situations) and nine experiment conditions with three different time delay issues.

	No time delay (NO)	Regular time delay (RE)	Irregular time delay (IR)	The number of invalid system performances (trials)	Ratio of invalid system performances (%)
Perfect System (PF)	NOPF^1)^	REPF^2)^	IRPF^3)^	0 out of 60 trials	0%
Expected Uncertainty situations (EX)	NOEX^4)^	REEX^5)^	IREX^6)^	30 out of 60 trials	50%
Unexpected Uncertainty situations (UX)	NOUX^7)^	REUX^8)^	IRUX^9)^	6 out of 60 trials	10%

NOPF^1)^: no time delay under the perfect system; REPF^2)^: regular time delay under the perfect system; IRPF^3)^: irregular time delay under the perfect system; NOEX^4)^: no time delay under the expected uncertainty situations; REEX^5)^: regular time delay under the expected uncertainty situations; IREX^6)^: irregular time delay under the expected uncertainty situations; NOUX^7)^: no time delay under the unexpected uncertainty situations; REUX^8)^: regular time delay under the uncertainty situations; IRUX^9)^: irregular time delay under the uncertainty situations.

The perfect system situations, such as no time delay under the perfect system (NOPF), the regular time delay under the perfect system (REPF), and the irregular time delay under the perfect system (IRPF), have a 0% ratio of invalid system performance (i.e., 0 invalid system performance out of 60 decision-making trials) as most reliable system usage environment for decision-making.

The expected uncertainty situations, such as no time delay under the unexpected uncertainty situations (NOEX), the regular time delay under the expected uncertainty (REEX), and the irregular time delay under the expected uncertainty situations (IREX), have a 50% ratio of invalid system performance (i.e., 30 invalid system performances out of 60 decision-making trials) as unreliable system usage environment for decision-making.

The unexpected uncertainty situations, such as no time delay under the unexpected uncertainty situations (NOUX), the regular time delay under the unexpected uncertainty (REUX), and the irregular time delay under the unexpected uncertainty situations (IRUX) have a 10% ratio of invalid system performance (i.e., six invalid system performances out of 60 decision-making trials) as a reliable system usage environment for decision-making.

### 2.3 Electroencephalogram instrumentation

We used EEG to understand the cognitive process based on decision-making in a multiple-cue judgment system. EEG measures human electrical signals as brain activity from the scalp. The cognitive process is interpreted by changes in electrical signals when humans experience specific stimuli. The electrical signal data is measured and collected by electrodes (channel). We used a 10–20 system. The common mode sense (CMS) and driven right leg (DRL) are located, respectively, AFz and FCz, and two mastoid electrodes (TP9 and TP10) are offline re-referenced to improve EEG quality (Fong et al., 2012; Stopczynski et al., 2014). Thus, 30 electrodes were used (Fp1, Fp2, F7, F3, Fz, F4, F8, FC5, FC1, FC2, FC6, T7, C3, Cz, C4, T8, CP5, CP1, CP2, CP6, P7, P3, Pz, P4, P8, PO9, O1, Oz, O2, and PO10) for EEG data analysis. The EEG device was an Emotiv EpocFlex get kit, and the data sampling size was 128 Hz.

### 2.4 Demographic information of participants

The experiment was conducted with 19 participants. The participants’ ethnicity was 11 Africans, two African Americans, three Asians, and two Caucasian Americans. The average age is 32.1, and all participants are older than 18. All participants had no physical or psychological disorder to conduct experiments. For every experiment session, we asked whether they needed a rest. The average experiment time is 63.85 min. This study proceeded with institutional review board (IRB) approval with received consent from all participants.

### 2.5 Electroencephalogram data preprocessing

Data preprocessing is necessary for EEG data because of noise and artifacts, such as eye or muscle movement, in raw EEG data. The EEG data were filtered by the higher edge of the frequency at 30 Hz and epoched before 200 ms to after 1,000 ms from the onset as the timing when participants see the feedback after the decision-making. We referenced EEG data based on two mastoids (TP9 and TP10), and the baseline was removed before 200 ms to remove noise and normalize EEG data (Fong et al., 2012). We used independent component analysis (ICA) to remove artifacts and only retained brain activities after ICA. Finally, we removed EEG epochs when the voltage is higher than 75 μV or lower than −75 μV to remove EEG data having extremely high or low voltage. We used EEGLAB (v2022.1) as an EEG data preprocessing and analysis software tool based on MATLAB for EEG data preprocessing. ERP analysis was performed according to ERP epochs for nine conditions (3 uncertainty situations × 3 time delay conditions), as shown in Table 1.

### 2.6 Statistical analysis

For significant analysis of the effect of uncertainty situations and time delay issues, we conducted an analysis of variance (ANOVA) by using the SAS program (Version 9.41) for ERP amplitude. The dependent variables are uncertainty situations (EX, PF, and UX) and time delay conditions (IR, NO, and RE). The significance level is 0.05 (α < 0.05). For the post-hoc test, we used Tukey’s test.

## 3 Results

### 3.1 Data visualization of event-related potential analysis

**Figure 6:**
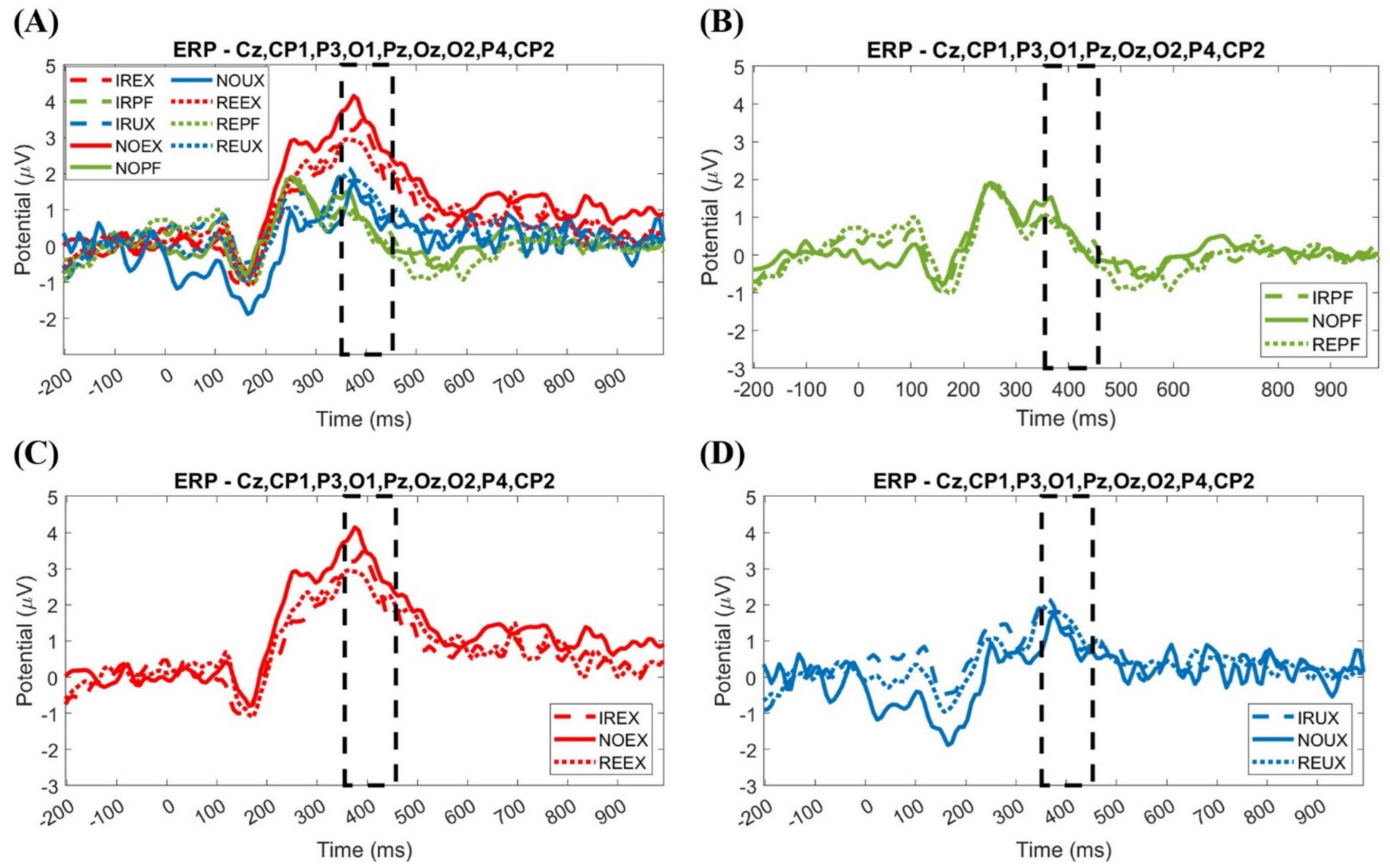
Event-related potential plot for nine conditions and system usage environments. **(A)** 9 conditions; **(B)** Perfect systems (PF); **(C)** Expected uncertainty situations (EX); **(D)** Unexpected uncertainty situations (UX).

We used the ERP analysis to understand how reliable or unreliable system usage environment and time delay affect human decision-making. ERP is the interpretation of change in recorded electrical signal data based on the time domain from the onset when humans see or experience specific stimuli. The EEG analysis of this study shows a P300 (or P3) pattern showing a positive peak amplitude (potential value) of approximately 300 ms. The time range is 350–450 ms for each of the nine conditions around the parietal lobe part with six channels (Cz, CP1, P3, O1, Pz, Oz, O2, P4, and CP2) based on Pz. Also, P300 patterns are shown in terms of system usage environment by systems usage reliability, as shown in Figure 6.

For eye inspection of the ERP plot in Figure 6, the grand averaged ERP plot shows positive peaking approximately 300 ms (350 ~ 450 ms) after onset. In the ERP plot, the expected uncertainty situations (IREX, NOEX, and REEX) seem to have the highest amplitude rather than the unexpected uncertainty situations (IRUX, NOUX, and REUX) and perfect system situations (IRPF, NOPF, and REPF). In the time delay conditions, no delay seems to have the highest amplitude in the expected uncertainty situation and perfect system.

**Figure 7:**
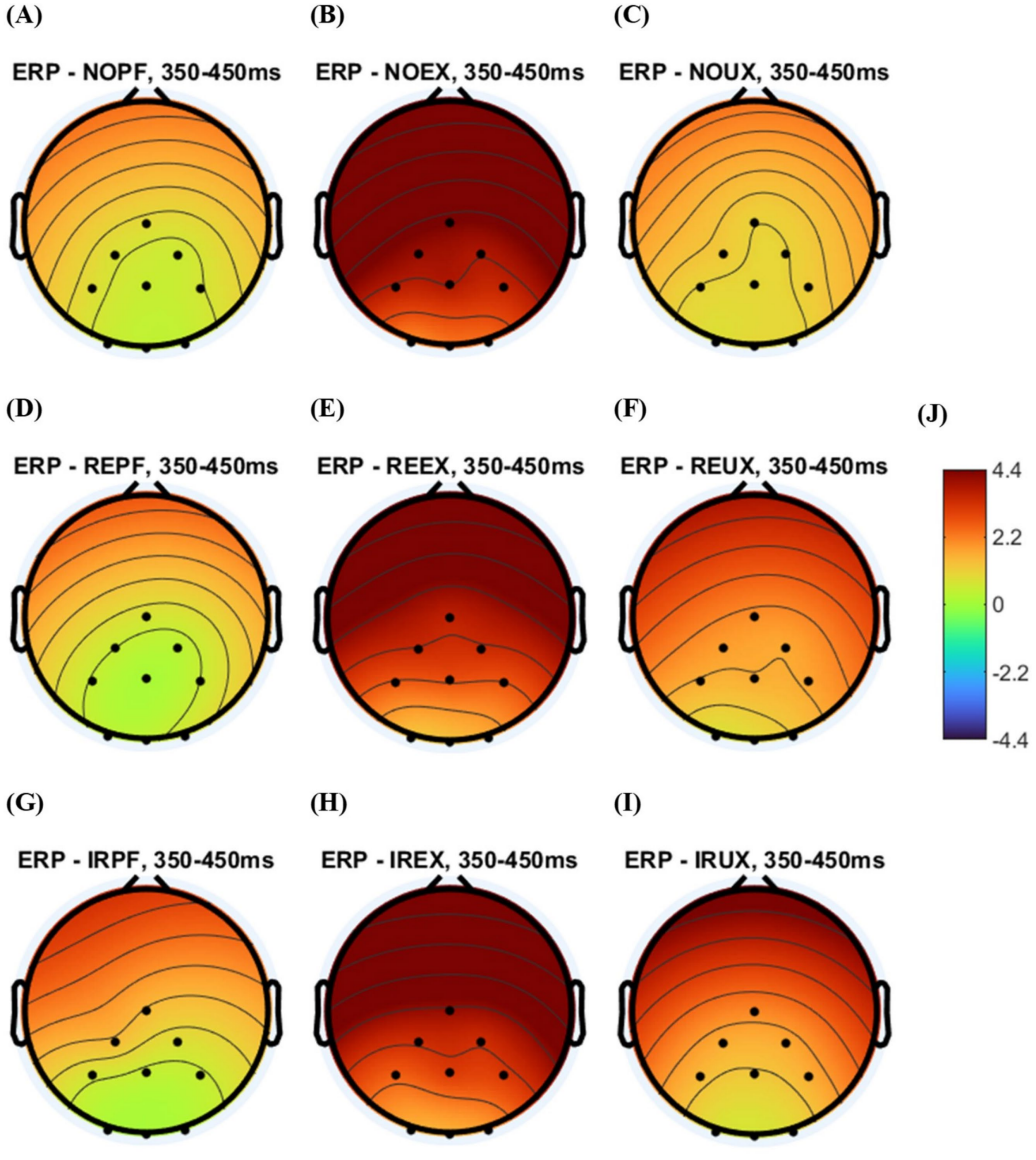
The grand averaged topography plot by system usage environment and time delay conditions: **(A)** no time delay under perfect system (NOPF); **(B)** no time delay under the expected uncertainty situations (NOEX); **(C)** no time delay unexpected under the unexpected uncertainty situations (NOUX); **(D)** regular time delay under perfect system (REPF); **(E)** regular time delay under the expected uncertainty situations (REEX); **(F)** regular time delay under the unexpected uncertainty situations (REUX); **(G)** irregular time delay under perfect system (IRPF); **(H)** irregular time delay under the expected uncertainty situations (IREX); **(I)** irregular time delay under the unexpected uncertainty situations (IRUX); **(J)** potential value (amplitude) range.

We plotted EEG data as averaged topography to know how different system performance environments change brain activity and time delay conditions by showing averaged potential values. The positivity’s tone is warm, and the color becomes thicker when the potential value increases, as shown in Figure 7. Positivity means having an area of positive amplitude (microvolts).

In visual inspection, the positivity of NO, RE, and IR conditions seem to have similar positivity in the expected uncertainty (EX) (see Figures 7B,E,H) and perfect system (PF) (see Figures 7A,D,G). In the unexpected uncertainty (UX) situation, NO conditions look to have the smallest positivity (see Figure 7C).

Table 2 shows the grand averaged amplitude by system usage environment and time delay conditions (nine conditions). Table 2 also shows the mean of the grand average by three different system usage environments (uncertainty situations) and three different time delay types.

**Table 2:** The grand averaged amplitude is determined by the system usage environment and time delay conditions.

	Perfect system (PF)		Expected uncertainty (EX)		Unexpected uncertainty (UX)		Mean of grand averaged amplitude by NO/RE/PF	
No time delay (NO)	NOPF^1)^	0.6958	NOEX^2)^	3.401	NOUX^3)^	1.059	NOPF & NOEX & NOUX	1.7186
Regular time delay (RE)	REPF^4)^	0.518	REEX^5)^	2.57	REUX^6)^	1.532	REPF & REEX & REUX	1.5402
Irregular time delay (IR)	IRPF^7)^	0.6811	IREX^8)^	2.804	IRUX^9)^	1.412	IRPF & IREX & IRUX	1.6322
Mean of grand averaged amplitude by EX/PF/UX	NOPF & REPF & IRPF		NOEX & REEX& IREX		NOUX & REUX& IRUX			
	0.6317^b10)^		2.9248^a^		1.3344^b^			

Three different system usage environments (uncertainty situations) and three different time delay types (unit: microvolts (μV)). NOPF^1)^: no time delay under the perfect system; NOEX^2)^: no time delay under the expected uncertainty situations; NOUX^3)^: no time delay under the unexpected uncertainty situations; REPF^4)^: regular time delay under the perfect system; REEX^5)^: regular time delay under the expected uncertainty situations; REUX^6)^: regular time delay under the unexpected uncertainty situations; IRPF^7)^: irregular time delay under the perfect system; IREX^8)^: irregular time delay under the expected uncertainty situations; IRUX^9)^: irregular time delay under the unexpected uncertainty situations; ^10)^ means of the grand averaged amplitude with different letters in a row are different at 5% significance level by Tukey’s test by three uncertainty situations of nine conditions.

In grand averaged amplitude, no time delay under expected uncertainty shows the highest amplitude (3.021 μV). We used two-way ANOVA for P300 potential values (amplitude) in nine conditions. The different uncertainty situations significantly affected P300 amplitude differences (*F* = 7.76, *p* = 0.0006 < 0.05). The grand averaged P300 amplitude was calculated for the parietal lobe part channels (Cz, CP1, P3, O1, Pz, Oz, O2, P4, and CP2) for 350–450 ms. The means of amplitude by uncertainty situations and time delay conditions is the mean of the grand averaged P300 amplitude value. In Tukey’s test for the post-hoc test, the expected uncertainty situation makes a significant amplitude difference from other situations, such as perfect system (*p* = 0.0004) and unexpected uncertainty situations (*p* = 0.0203). However, there is no significant difference in amplitude change between unexpected uncertainty situations and a perfect system (*p* = 0.4568). In time delay conditions, different time delay conditions did not significantly affect P300 amplitude differences (*F* = 0.04, *p* = 0.9562). Also, there is no interaction effect between uncertainty situations and time delay conditions (*F* = 0.22, *p* = 0.9297). It means these two dependent variables have no relationship to affect amplitude change.

### 3.2 Event-related potential analysis by three uncertainty situations

**Figure 8:**
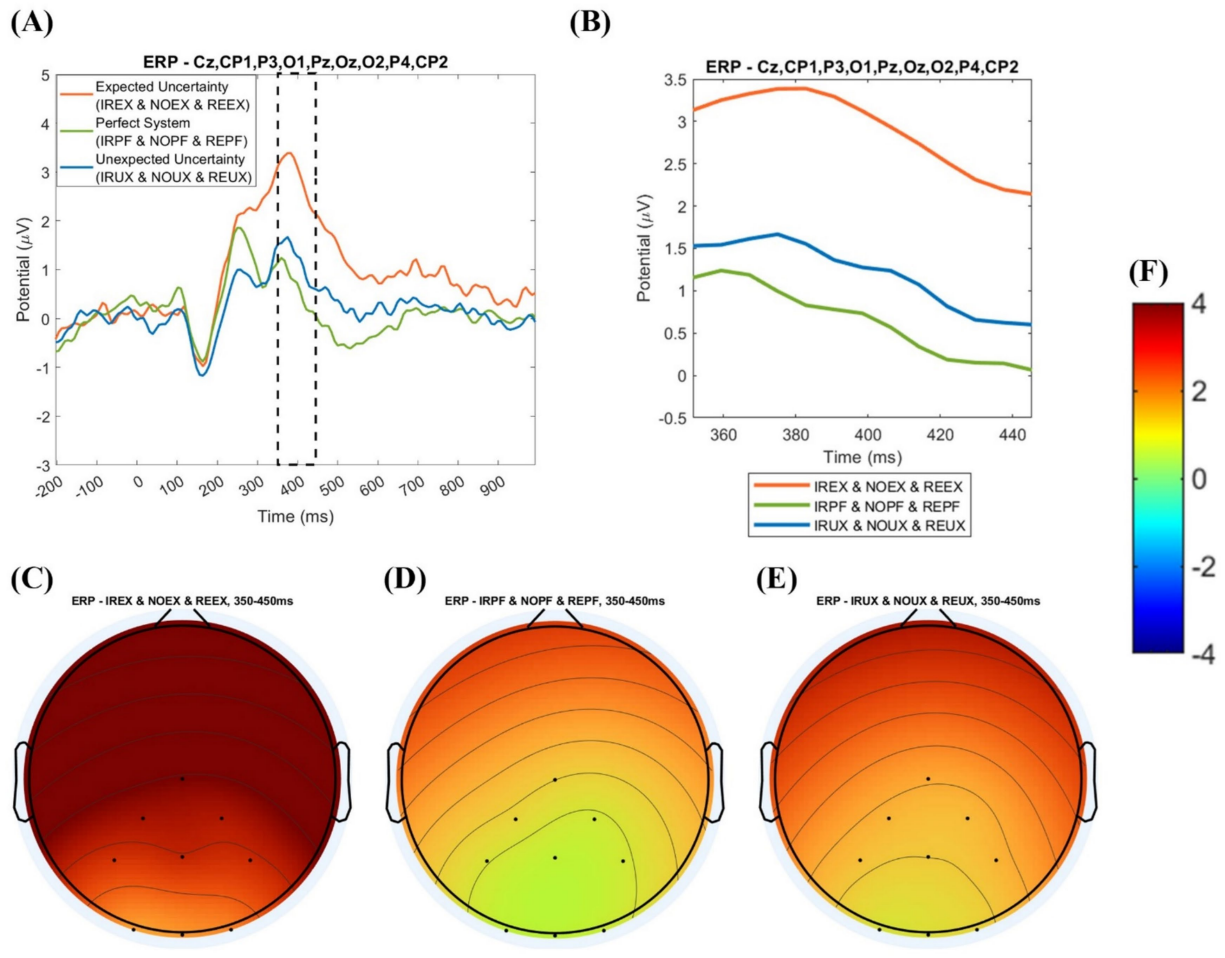
Event-related potential analysis by three time delay conditions with amplitude values: **(A)** grand averaged event-related potential plot by uncertainty situations (−200 to 1,000 ms); **(B)** grand averaged event-related potential plot uncertainty situations (350–450 ms); **(C)** topography of the expected uncertainty situations (EX); **(D)** topography of perfect system (PF); **(E)** topography of the unexpected uncertainty situations (UX); **(F)** potential value (amplitude) range.

Figure 8 shows the grand averaged ERP analysis by three uncertainty situations. There is a difference between the nine-condition analysis and the three uncertainty situations analysis.

In the nine-condition analysis, the mean of grand averaged amplitude was calculated for the 19 subjects for uncertainty situations. The analysis is visualized in a grand averaged ERP plot before 200 to after 1,000 ms from the onset in Figure 8A, 350–450 ms from the onset, and topography with grand averaged amplitude in Figure 8B.

In visual inspection, EX shows the largest positivity (see Figure 8C), and PF shows the smallest (see Figure 8D). In grand averaged potential values by system usage environments, such as the grand average of no time delay, regular time delay, and irregular time delay under the uncertainty environment, the expected uncertainty environment (the grand average of NOEX, REEX, and IREX environments) shows the highest potential value (2.903 μV). The unexpected uncertainty environment (the average of NOUX, REUX, and IRUX) shows the second-highest amplitude (1.196 μV). The perfect system environment (the average of NOPF, REPF, and IRPF) shows the lowest amplitude (0.6439 μV), as shown in Table 3.

**Table 3:** The grand averaged ERP amplitude for three uncertainty situations from 350 to 450 μV.

	Expected uncertainty situation (EX)	Perfect system situation (PF)	Unexpected uncertainty situation (UX)
Amplitude (μV)	2.903^a1)^	0.6439^b^	1.196^ab^

^1)^Means of the grand averaged amplitude with different letters in a row are different at 5% significance level by Tukey’s test by three uncertainty situations of nine conditions.

We performed a one-way ANOVA to determine the significant differences in the grand averaged P300 amplitude under uncertainty situations. The amplitude size is significantly different in uncertain situations (*F* = 3.34, *p* = 0.0429). In the Tukey post-hoc test, the perfect system and the expected uncertainty situations have significant amplitude differences (*p* = 0.0425). In contrast, expected and unexpected uncertainty situations have no significant amplitude differences (*p* = 0.1564). Also, the perfect system and unexpected uncertainty situations have no significant difference (*p* = 0.8173).

### 3.3 Event-related potential analysis by three time delay conditions

**Figure 9:**
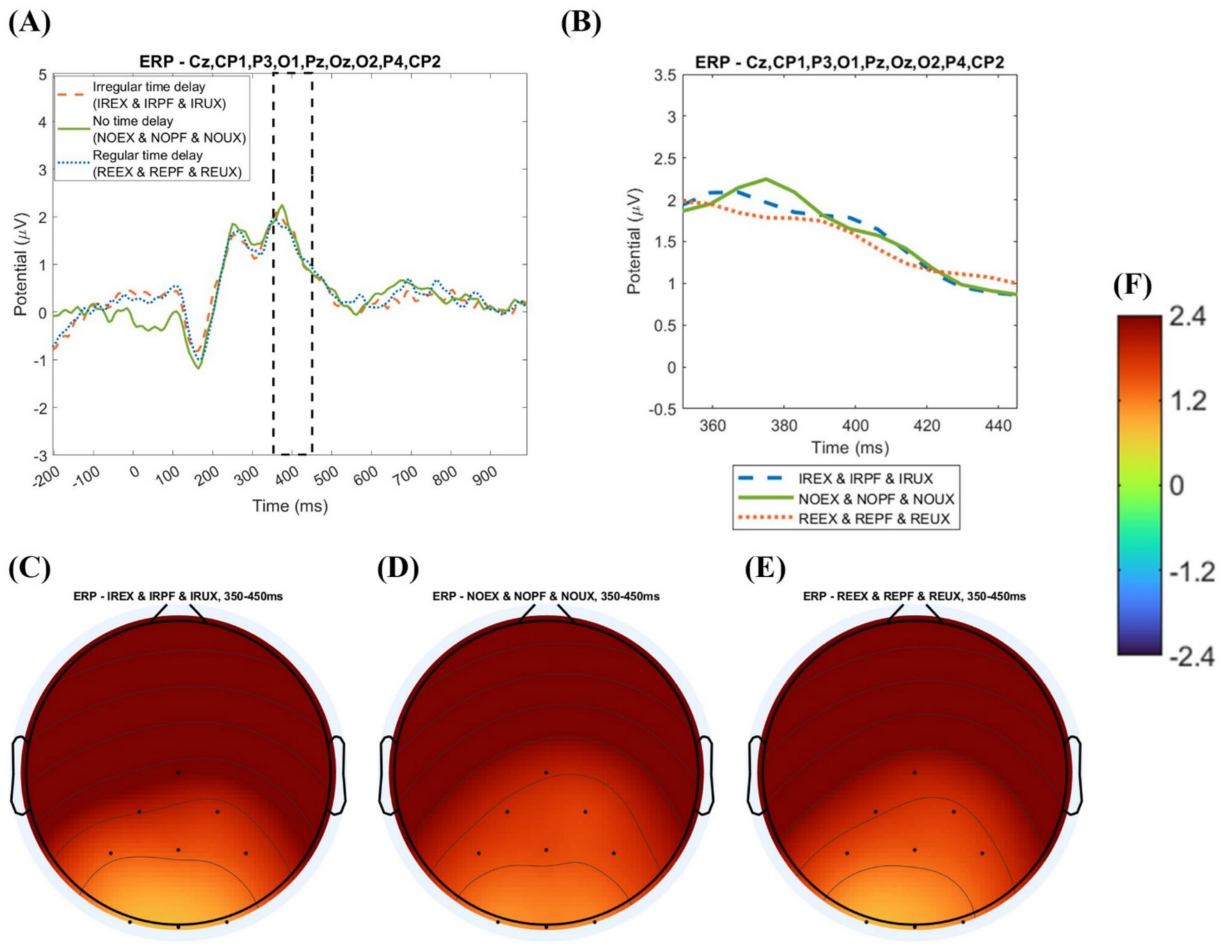
Event-related potential analysis by three time delay conditions with amplitude values: **(A)** grand averaged event-related potential plot by time delay (−200 to 1,000 ms); **(B)** grand averaged event-related potential plot by time delay (350–450 ms); **(C)** topography of irregular time delay (IR); **(D)** topography of no time delay (NO); **(E)** topography of regular time delay (RE); **(F)** potential value (amplitude) range.

Figure 9 shows the ERP analysis by three time delay conditions. The analysis is visualized in the grand averaged ERP plot before 200 ms to after 1,000 ms from the onset, 350–450 ms from the onset, and topography with grand averaged amplitude.

In averaged potential values by time delay conditions, such as the grand average of the perfect system, expected uncertainty, and unexpected uncertainty environments with no time delay, the no time delay conditions (the grand average of NOPF, NOEX, and NOUX conditions) show the highest potential value (1.592 μV). The grand average of irregular time conditions (the grand average of IRPF, IREX, and IRUX conditions) shows the second-highest potential value (1.568 μV). The average of regular time conditions (the grand average of REPF, REEX, and REUX conditions) has the lowest potential value (1.514 μV), as shown in Table 4. In one-way ANOVA in time delay conditions, there is no significant amplitude difference by different time delay conditions (*F* = 0.00, *p* = 0.9958).

**Table 4:** The grand averaged ERP amplitude for three time delay conditions from 350 to 450 μV.

	No time delay (NO)	Irregular time delay (IR)	Regular time delay (RE)
Amplitude (μV)	1.592	1.568	1.514

## 4 Discussion

The P300 pattern can be interpreted as memory load. Memory load (or working memory load) is the cognitive ability to temporarily store and use information to support decision-making (Baddeley, 2003; Cowan, 1999; Scharinger et al., 2017). High memory load makes impairment of cognitive reappraisal of emotional responses for specific situations (Gan et al., 2017). In memory load, positive peaking or deflection for 300–600 ms after onset (Kok, 1997; Kramer et al., 1986; Mecklinger et al., 1992). Wang et al. (2015) set up 450–550 ms in terms of memory load. Thus, 350–450 ms is a reasonable time window to know the impact of expected and unexpected uncertainty in memory load. In studies on memory load with P300, memory load can be increased when P300 amplitude is decreased (Wang et al., 2015).

### 4.1 Memory load by nine conditions

**Figure 10:**
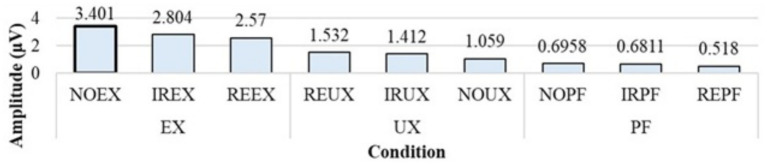
Comparison graph for the mean of grand averaged P300 amplitude from 350 to 450 ms under nine conditions.

The no time delay under the expected uncertainty condition (NOEX) shows the highest amplitude (3.021 μV), as shown in Figure 10.

This result can be interpreted as the memory load being reduced in the unreliable system usage environment (expected uncertainty situation) when information delivery has no time delay. In terms of uncertainty situations, the expected uncertainty situations (NOEX, IREX, and REEX) show the highest amplitude values. The unexpected uncertainty situations (REUX, IRUX, and NOUX) show the second-highest amplitude values. Finally, the lowest amplitude is shown in the perfect system situations (NOPF, IRPF, and REPF).

In two-way ANOVA, there is a significant amplitude change by the uncertainty situations (*p* = 0.0006). There is also a significant amplitude difference between the expected uncertainty and two other uncertainty situations. The expected uncertainty situations showed a higher mean of grand averaged P300 amplitude (2.9248 μV) than two other uncertainty situations, such as the unexpected uncertainty situation (1.3344 μV) and perfect system (0.6317 μV), as shown in Table 2. This means that an unreliable system usage environment can cause a less memory load that shows a higher P300 amplitude than a reliable system usage environment.

### 4.2 Memory load under reliable or unreliable system usage environment

The expected uncertainty environment shows the highest amplitude (2.903 μV) when compared with the other two system usage environments, such as unexpected uncertainty (1.196 μV) and the perfect system (0.6439 μV) environments, as shown in Table 3. In Tukey’s post-hoc analysis after one-way ANOVA analysis, there is a significant difference in amplitude between the expected uncertainty situation and the perfect system (*p* = 0.0425). In contrast, there are no significant differences between expected and unexpected uncertainty situations (*p* = 0.1564) or between unexpected uncertainty situations and perfect systems (*p* = 0.8173).

In two-way ANOVA for the mean of grand averaged P300 amplitude in nine conditions, the expected uncertainty situations and the other two are significantly different (*p* = 0.0004 for PF; *p* = 0.0203 for UX). The expected and perfect systems are significantly different in one-way ANOVA for grand averaged P300 amplitude in three uncertainty situations. The expected uncertainty situations (EX) show a higher P300 amplitude for the other two uncertainty situations. This means that expected uncertainty situations lower memory load. Based on this result, we can conclude that the observation of an invalid result, which affects unreliable environment construction by expected or unexpected uncertainty situations, requires less memory load.

The reason for showing high P300 amplitude under unreliable system usage would be that humans will not follow the decision support system by distrusting the unreliable system usage environment. The expected or unexpected uncertainty environment makes an unreliable environment, unlike a perfect system showing no invalid result after decision-making. The experience of invalid performance can make human agents not trust the decision support system and information because of invalid results, despite following system rules for decisions. By distrusting the systems, the human agents will make decisions without considering information from agents. In the perfect system, it shows no invalid result if they follow the decision policy of the multiple-cue judgment system. So, the perfect system shows valid performance when human agents follow system rules. For this reason, they need to remember the rules to get valid results with the memory load increase. Thus, it can be considered that unreliable system environments result in less remembering rules for utilizing the system due to distrustful experiences by expected or unexpected uncertainty, so a low memory load is shown in the multiple-cue judgment environments. However, we need to study the relationship between memory load and human behavior that does not follow the decision policy of the system.

### 4.3 Memory load under time delay issue

The NO condition shows the highest amplitude (1.592 μV) in the time delay issue. The IR condition shows the second-highest amplitude (1.568 μV), and the RE shows the lowest amplitude (1.514 μV) in Table 4. In two-way ANOVA and one-way ANOVA, there are no significant amplitude changes by time delay conditions (*F* = 0.00, *p* = 0.9958 in a one-way ANOVA; *F* = 0.04, *p* = 0.9562 in two-way ANOVA). Thus, it can be concluded that delayed information delivery does not affect memory load.

In the time delay aspect, the error-related negativity (ERN or Ne) patterns were shown in specific electrodes. ERN is shown during a 0–100-ms time window after onset. ERN can reflect cognitive states for mismatching expected and real-world results (Larson et al., 2010). In this study, there were ERN patterns during 0–100 ms. One of the ERN studies mentioned that this ERN amplitude is negatively increased when emphasizing the accuracy of matching between result and decision-making rather than emphasizing reaction time, such as notifying “your decision is slow” (Mattes et al., 2023). Based on this study, the time delay aspect can be interpreted as pressure on accuracy or reaction time for decision-making. Also, we will approach different analysis methods, such as frequency domain analysis, to find how the cognitive process operates by delayed information delivery.

## 5 Conclusion

This study is about understanding the cognitive process under the reliability of system usage environment with environmental uncertainty and time delay issues in multiple-cue judgment systems, such as MASs. We used the EEG for the neurological analysis of this study. The EEG analysis shows that P300 appears, and we interpret this result in terms of memory load. We can conclude that the memory load is significantly reduced under unreliable system usage environments because the decision policy of a system is less considered when making decisions to use the system. In this case, we need to perform a study to understand how memory load changes when humans do not follow the decision policy of the system for decision-making. In the time delay issue in information delivery, we could find that delayed information delivery does not significantly affect memory load for decision-making in multiple-cue judgment systems.

The significant finding of this study is about differences in memory load by unreliable or reliable system usage environment (expected or unexpected uncertainty situations), not the measurement of cognitive states by valid or invalid feedback, as prior studies have been studied. However, the participants of the ethnicity group were imbalanced, so there is a weakness in normalized analysis by specific ethnicity groups. Also, this study proceeded with a theoretical approach to the visual aspect of the user interface. For practical analysis of cognitive states, the practical experiment can be conducted using a practical user interface for a specific automated system. The time delay of information delivery will be conducted with another ERP pattern, such as ERN, to interpret cognitive states. Also, we will perform other domain analyses for EEG data, such as frequency domain analysis, to determine which cognitive process operates due to delayed information delivery.

This study is about understanding cognitive states in an unreliable MAS usage environment with multiple sources of information. This study focuses on an unreliable system usage environment, which is not a valid (good) or invalid (poor) system performance. This unique study aspect can give insight into measuring and monitoring human cognitive states and whether users are trapped in unreliable MAS. Thus, we expect insight into an index of categorization of neural states under a reliable or unreliable decision-making environment to use a multiple-cue judgment system with information delivery delays from this study.

## Data Availability

The raw data supporting the conclusions of this article will be made available by the authors, without undue reservation.
